# BKM120 induces apoptosis and inhibits tumor growth in medulloblastoma

**DOI:** 10.1371/journal.pone.0179948

**Published:** 2017-06-29

**Authors:** Ping Zhao, Jacob Hall, Mary Durston, Austin Voydanoff, Elizabeth VanSickle, Shannon Kelly, Abhinav B Nagulapally, Jeffery Bond, Giselle Saulnier Sholler

**Affiliations:** 1 Pediatric Oncology Translational Research Program, Helen DeVos Children’s Hospital, Grand Rapids, MI, United States of America; 2 Calvin College, Grand Rapids, MI, United States of America; 3 College of Human Medicine, Michigan State University, Grand Rapids, MI, United States of America; University of South Alabama Mitchell Cancer Institute, UNITED STATES

## Abstract

Medulloblastoma (MB) is the most common malignant brain tumor in children, accounting for nearly 20 percent of all childhood brain tumors. New treatment strategies are needed to improve patient survival outcomes and to reduce adverse effects of current therapy. The phosphatidylinositol-3-kinase (PI3K)/AKT/mammalian target of rapamycin (mTOR) intracellular signaling pathway plays a key role in cellular metabolism, proliferation, survival and angiogenesis, and is often constitutively activated in human cancers, providing unique opportunities for anticancer therapeutic intervention. The aim of this study was to evaluate the pre-clinical activity of BKM120, a selective pan-class I PI3K inhibitor, on MB cell lines and primary samples. IC_50_ values of BKM120 in the twelve MB cell lines tested ranged from 0.279 to 4.38 μM as determined by cell viability assay. IncuCyte ZOOM Live-Cell Imaging system was used for kinetic monitoring of cytotoxicity of BKM120 and apoptosis in MB cells. BKM120 exhibited cytotoxicity in MB cells in a dose and time-dependent manner by inhibiting activation of downstream signaling molecules AKT and mTOR, and activating caspase-mediated apoptotic pathways. Furthermore, BKM120 decreased cellular glycolytic metabolic activity in MB cell lines in a dose-dependent manner demonstrated by ATP level per cell. In MB xenograft mouse study, DAOY cells were implanted in the flank of nude mice and treated with vehicle, BKM120 at 30 mg/kg and 60 mg/kg via oral gavage daily. BKM120 significantly suppressed tumor growth and prolonged mouse survival. These findings help to establish a basis for clinical trials of BKM120, which could be a novel therapy for the treatment of medulloblastoma patients.

## Introduction

Although improvements in the past 40 years have led to markedly improved survival rates of approximately 80% overall for pediatric cancers, patients with relapsed, rare and advanced stage tumors still have a very poor prognosis. Medulloblastoma (MB) is a highly malignant primary brain tumor that originates in the cerebellum or posterior fossa. It is the most common malignant brain tumor in pediatric patients, accounting for 20% of all brain tumors in children [1]. The current standard of care for patients with MB aged 3 years or older involves surgery followed by craniospinal radiation and chemotherapy [2]. Five-year event-free survival rates for patients with high-risk MB are >60% and can be >80% in patients with standard-risk disease [2, 3]. Recurrence and progression of disease occurs in 15–20% of standard risk and 30–40% of high risk patients, resulting in poor survival outcome [4]. High neurocognitive burden is associated with current treatments and more specifically with radiotherapy in survivors [5]. Therefore, new treatment strategies are needed for control of MB. There is a growing focus on treating MB based on the biology of the diseases rather than with “one size fits all” therapy.

Medulloblastoma is increasingly recognized as a heterogeneous disease with histopathological and molecular variants that have distinct biological behaviors. It may occur in a context suggestive of a Li Fraumeni syndrome (*TP53* mutations) but no recurrent cancer gene mutations have been found [6, 7]. Nevertheless, many of the mutations described so far affect key intracellular signaling pathways such as sonic hedgehog, Wnt/β-catenin and Phosphoinositide-3-Kinase (PI3K/AKT/mTOR) pathways. The PI3K/AKT/mTOR pathway plays a key role in cellular metabolism, proliferation, survival, angiogenesis and migration [8–10]. This pathway is often constitutively activated in human tumor cells, providing unique opportunities for anticancer therapeutic intervention [11]. Several agents that target PI3K/AKT pathway are currently in clinical development, including mTOR inhibitors, PI3K inhibitors (pan-class I and isoform specific), dual PI3K/mTOR inhibitors, and AKT inhibitors [12, 13]. The PI3K/AKT signaling pathway has often been reported to be deregulated in MB, with numerous genetic alterations involving this network occurring independently of the particular subtype [14]. Strategies that are currently being evaluated with targeted agents against this pathway in MB were summarized by Dimitrova and Arcaro [7].

BKM120 (Buparlisib) is an oral pan-class I PI3K inhibitor that targets all four catalytic isoforms of class I PI3K (p110α, p110β, p110δ and p110γ). Targeting PI3K with BKM120 decreases PI3K/AKT/mTOR signaling and has anti-proliferative, pro-apoptotic, and anti-angiogenic effects in preclinical models [15]. BKM120 is currently being clinically evaluated for the treatment of different adult cancers including advanced solid tumors, breast cancer, prostate cancer, advanced non-small cell lung cancer, and colorectal cancer [16–21]. Since the blood-brain-barrier limits availability of drugs to the central nervous system (CNS), brain tumors are generally not considered attractive malignancies for initial drug screenings. However, BKM120 penetrates the blood-brain-barrier, and clinical evaluations in glioblastoma are being conducted [22, 23]. While a few studies have examined a role of BKM120 in medulloblastoma, [24–26] no clinical studies have been reported. A thorough understanding of BKM120 mode of action and molecular studies are required to rationally design clinical studies of BKM120 for the treatment of medulloblastoma. In this preclinical study, we screened the feasibility of BKM120 as a novel drug treatment in MB using established and patient primary MB cell lines. Our genomic profiling analysis, *in vitro* and *in vivo* studies have provided strong evidence for an anti-tumor activity of BKM120 and a clinical relevance of this compound in the treatment of MB.

## Materials and methods

This study was carried out in strict accordance with the recommendations in the Guide for the Care and Use of Laboratory Animals of the National Institute of Health. The protocol was approved by the Committee of the Ethics of Animal Experiments of the Spectrum Health and Calvin College. Injection to mice was performed using aseptic technique. Tumor volumes were measured by caliper; tumors were harvested post euthanasia of the animals. All efforts were made to minimize suffering.

### Compounds, reagents, and cell lines

BKM120 was provided by MTA from Novartis Pharma (Basel, Switzerland), and was prepared as 10 mmol/L stock solutions in 100% dimethyl sulfoxide (DMSO). Working solutions were freshly prepared before addition to the cell media. Dulbecco's Modified Eagle's Media (DMEM) and Fetal Bovine Serum (FBS) were purchased from Life Technologies. MB cell lines DAOY, D283 and D341 were purchased from ATCC (Manassas, VA). MB cell lines D384, D458, D487, D556, D581, and D721 were kindly provided by Dr. Darell Bigner at Duke University Medical Center (Durham, NC). BIO-054-08, BIO-074-08 and BIO-094-08 cells were primary cells from MB patients enrolled in the Signature’s study conducted at Helen DeVos Children’s Hospital in Grand Rapids, Michigan with the approval of the Institutional Review Board.

### RNA expression profiling analysis and DNA mutation analysis

RNA expression analysis was performed for all MB cells. 5 x 10^4^ cells were collected into 1.5 mL eppendorf tubes and centrifuged at 14,000 rpm for 10 minutes at 4°C to pellet. Pellets were rinsed twice with 500 μL of ice cold PBS with 1 μL SuperaseIn RNase inhibitor (Ambion, Austin, TX) before adding 250 μl of ice cold PBS with 2 μL SuperaseIn (Ambion) and storing at -80°C. All samples were shipped to Clinical Research Laboratories (Lenexa, KS) where Affymetrix GeneChip U133 Plus 2.0 genome wide expression cDNA microarray was used to determine RNA expression. All cell lines were tested in triplicate. All analysis was done using R/Bioconductor packages and Partek Genomics Suite. DNA mutation analysis was performed by Ion torrent sequencing. Data was analyzed with Torrent Suite Variant Caller.

### Cell viability and IC_50_ values

CellTiter-Glo Luminescent Cell Viability Assay (Promega, Madison, WI) was used to determine cell viability in 96-well plates. Cells were plated at 3,000 to 8,000 cells/well. On the next day cells were treated with increasing concentrations (0–4 μM) of BKM120 in DMEM with 10% FBS. DMSO in DMEM was used as vehicle control. After incubation with drug for 48 hrs, equal volume of CellTiter-Glo reagent (lysis buffer mixed with CellTiter-Glo substrate) was added to each well. Plates were rocked on an orbital shaker for 3 minutes at 20°C to induce cell lysis, and then were incubated an additional 10 minutes at 20°C to allow luminescent signal stabilization. Luminescence was measured with a BioTek plate reader and Gen5 software (BioTek Instruments Inc, Winooski, VT). IC_50_ values were calculated with a four-parameter variable-slope dose response curve using GraphPad Prism v.5 software.

### Cytotoxicity and apoptosis assays

IncuCyte ZOOM Live-Cell Imaging system (Essen Bioscience, Ann Arbor, MI) was used for kinetic monitoring of cytotoxicity and apoptotic activity of BKM120 in MB cells. Cells were seeded at 3,000 to 8,000 cells/well in 96-well black-walled plates. Cells were treated with increasing concentrations (0–4 μM) of BKM120 in DMEM with 10% FBS in the presence of 5 μM of Caspase 3/7 Apoptosis Assay Reagent (Essen Bioscience). The Caspase 3/7 reagent labels dead cells yielding green fluorescence. The plate was scanned and fluorescent and phase-contrast images were acquired in real time every 4 hours from 0 to 48 hours post treatment. Normalized Green Object Count per well at each time point and quantified time-lapse curves were generated by IncuCyte ZOOM software. Ratios of caspase 3/7 level in BKM120 treated cells compared to vehicle were plotted in Microsoft Excel.

### Western blot analysis

Cells were plated at 100,000 cells/well in 6-well plates and were allowed to adhere overnight prior to treating with BKM120 in DMEM with 10% FBS for 30 minutes, 1, 3, 24 or 48 hours. DMSO was used as vehicle control. Cells were lysed with RIPA lysis buffer (Cell Signaling, Danvers, MA), supplemented with a complete protease inhibitor (Thermo Fisher Scientific Inc., Waltham, MA). Cell lysates were collected and frozen at -80°C overnight to ensure complete cell lysis. Protein concentrations were determined by Bradford assay (Bio-Rad Laboratories, Hercules, CA). Lysates were electrophoresed on a 7.5% or 12% SDS-polyacrylamide gel in running buffer (Bio-Rad). Thirty micrograms of protein was loaded per lane. Gels were semi-dry transferred to nitrocellulose membranes using the Turbo Transfer system with Turbo Transfer buffer (Bio-Rad). Primary antibodies used were: rabbit polyclonal cleaved caspase 3 and caspase 3, rabbit polyclonal Phospho-AKT (Ser473), rabbit polyclonal AKT, rabbit monoclonal Phospho-S6 Ribosomal Protein (Ser235/236), rabbit monoclonal S6 Robosomal Protein, mouse monoclonal mTOR and rabbit monoclonal Phospho-mTOR, rabbit polyclonal β-actin, and rabbit polyclonal Vinculin (Cell Signaling). Goat anti-mouse or anti-rabbit HRP secondary antibody were used (Cell Signaling). Protein bands were detected using Clarity Western ECL Substrate (Bio-Rad).

### Measurement of cellular ATP level

CellTiter GLO luminescent assay (Promega), which measures total ATP levels, was combined with the CyQuant fluorescent DNA assay (Life Technologies, Grand Island, NY) to measure ATP level per cell. DAOY cells were plated at 3,000 cells/well, D283 and D458 cells were plated at 8,000 cells/well in 96-well black-walled plates. Cells were treated for 48 hrs with increasing concentrations (0–4 μM) of BKM120 in DMEM with 10% FBS. DMSO in DMEM was used as vehicle control. After 48 hrs, medium was aspirated and replaced with CellTiter-Glo reagent (lysis buffer mixed with CellTiter-Glo substrate, diluted with PBS 1:1) supplemented with CyQuant stock solution (50 μL dye per 10mL CellTiter-Glo reagent). Plates were rocked on an orbital shaker for 3 minutes at 20°C to induce cell lysis, and then were incubated an additional 10 minutes at 20°C to allow luminescent signal stabilization. Luminescence and fluorescence data were recorded using a BioTek plate reader and Gen5 software. CellTiter-Glo data were divided by CyQuant data and normalized to vehicle wells in order to generate data on ATP per cell.

### Mouse xenograft studies

*In vivo* drug studies were conducted with six-week-old female nude mice (nu/nu) from Charles River Laboratories (Portage, MI). Mice were housed in pathogen-free conditions and cared for in accordance with the Institutional Animal Care and Use Committee (IACUC) standards. Mice were injected with two million DAOY cells suspended in 100 μL of Matrigel (BD Biosciences, San Jose, CA) subcutaneously into the right flank. When tumors became palpable, mice were separated into vehicle control and BKM120-treated groups with the same average tumor volume, with 9 mice per group. BKM120 was dissolved in NMP/PEG300 (10/90 v:v) and administered to mice by oral gavage daily at 30 mg/kg and 60 mg/kg. Caliper tumor measurements were performed twice weekly. Tumor volume was determined by the equation length x width x depth x 0.52. Mice were followed to survival, each individual mouse was euthanized when its tumor reaches a maximum volume of 3 cm^3^.

### Statistics

ATP-per-cell assay was analyzed using multiple t-test between vehicle and BKM120 treatment using GraphPad Prism (La Jolla, CA). *In vivo* data was analyzed by two way anova to calculate significance among groups using R software.

## Results

### RNA expression levels of PI3K and mTOR in MB cell lines

**Fig 1:**
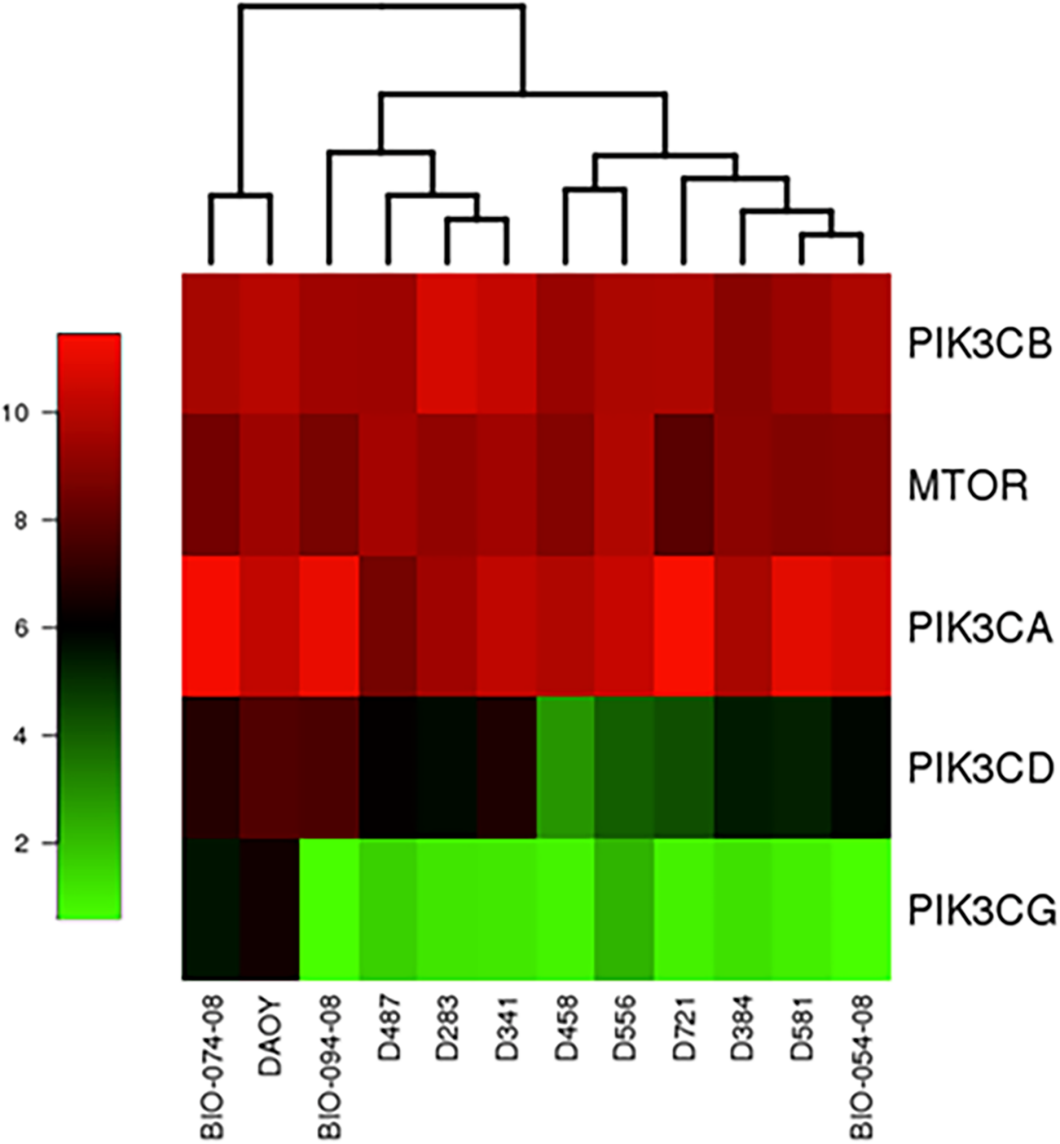
Genomic expression levels of PI3K in MB cells. Heat Map of RNA expression (MAS5 scores) of PI3K class I catalytic subunits and mTOR in MB cell lines.

BKM120 is an oral pan-class I PI3K inhibitor. We postulated that this compound will affect signaling molecules in PI3K/AKT/mTOR pathway and cell growth in MB. To examine the gene expression levels in PI3K/AKT/mTOR pathway of MB cell lines, Affymetrix GeneChip U133 Plus 2.0 genome wide expression cDNA microarray was used to determine RNA expression. All of our MB cell lines had high RNA expression levels of PI3K class IA catalytic isoforms (p110α, p110β, and p110δ encoded by the *PIK3CA*, *PIK3CB*, and *PIK3CD* genes respectively) and mTOR, indicated by MAS5 scores and Heat Map (Fig 1). Class IA is activated downstream of receptor tyrosine kinases (RTKs), while class IB (p110γ encoded by *PIK3CG*) is exclusively activated by G protein-coupled receptors. The PI3K p110α was reported to play a key role in the activation of the PI3K/AKT/mTOR pathway in MB [27].

### BKM120 exhibits cytotoxicity in a large panel of MB cell lines

**Fig 2:**
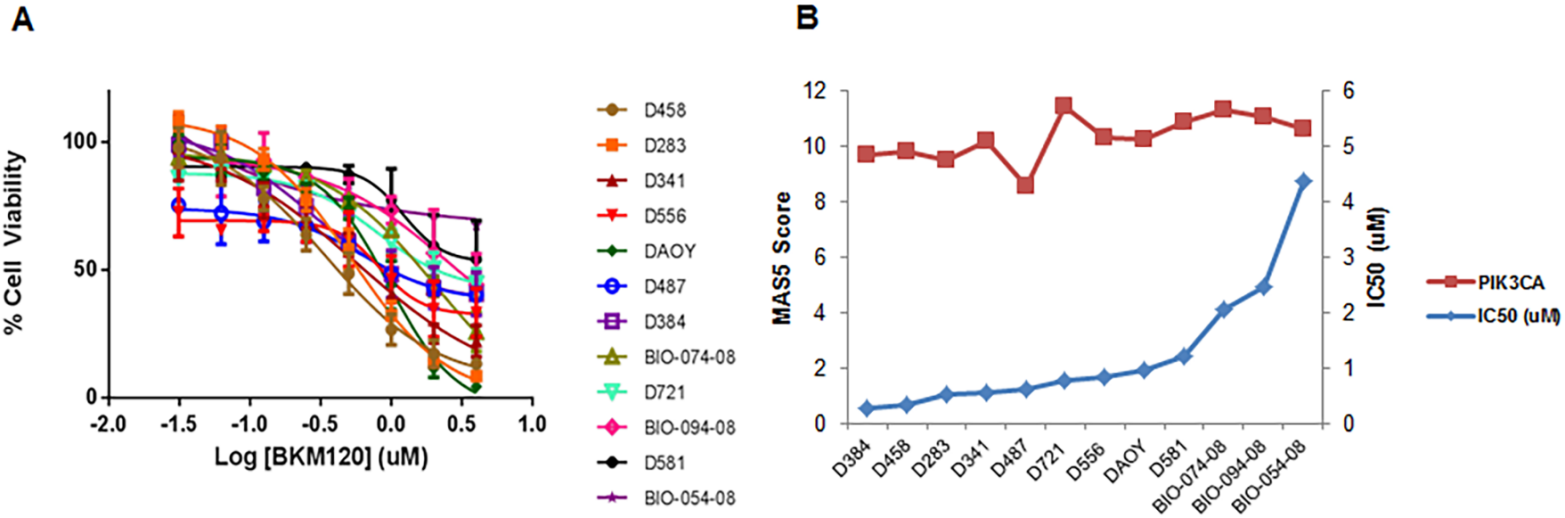
Evaluation of BKM120-mediated cytotoxicity in twelve MB cell lines. **(A)** MB cells were treated with BKM120 ranging from 31.25 nM to 4 μM over a course of 48 hours. Cell viability was determined by CellTiter-Glo assay. Percent cell viability was plotted and IC_50_ values were calculated by fitting the data to a four-parameter, variable slope dose-response model in GraphPad. Experiments were averages of three replicates. (B) PIK3CA gene expression MAS5 scores and BKM120 IC_50_ values in all MB cell lines.

BKM120 was examined in twelve MB cells for its cytotoxic efficacy. MB cells were treated with increasing concentrations of BKM120 ranging from 0 to 4 μM over a course of 48 hours. Cell viability at 48 hours post treatment was measured by CellTiter-Glo Luminescent Cell Viability Assay. Percent cell viability was plotted and IC_50_ values were calculated by fitting the data to a four-parameter, variable slope dose-response model in GraphPad. Data were compiled from three replicate experiments. All cell lines were sensitive to BKM120, with IC_50_ values that ranged from 0.279 to 4.38 μM (Fig 2A and Table 1). We did not observe any noticeable relationship between the sensitivity to BKM120 and PIK3CA expression levels in MB cell lines or primary cells (Fig 2B and Table 1). Cells with wild type *TP53* (D384, D458, D283, D341, D487, D721 and D556) showed moderately lower IC_50_ values as compared to cells harboring *TP53* mutation (DAOY and D581) (Table 1). We found that patient-derived primary cell lines (BIO-054-08, BIO-074-08 and BIO-094-08) were exceptions. They grew much slower in culture and were more resistant to BKM120 treatment.

**Table 1:** BKM120 IC_50_ values, PIK3CA gene expression MAS5 scores and *TP53* mutation status in MB cell lines.

Cell line	IC50 (μM)	PIK3CA	*TP53*
D384	0.2787	9.67549	WT
D458	0.345	9.8211	WT
D283	0.5159	9.49425	WT
D341	0.5644	10.1867	WT
D487	0.6242	8.58009	WT
D721	0.7866	11.4184	WT
D556	0.828	10.3277	WT
DAOY	0.9554	10.2347	Mutant
D581	1.214	10.9101	Mutant
BIO-074-08	2.066	11.3347	WT
BIO-094-08	2.459	11.0542	WT
BIO-054-08	4.375	10.6483	WT

### BKM120 induces apoptosis in MB cells in a dose and time-dependent manner

**Fig 3:**
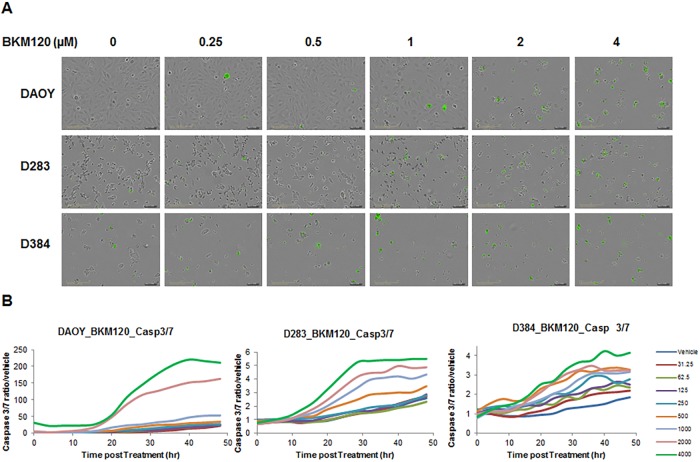
BKM120 induces apoptosis in MB cells in a dose and time-dependent manner. (A) Representative images of MB cells stained with Caspase 3/7 reagent at 48 hours post treatment of BKM120. (B) Real-time quantification of caspase 3/7 level in MB cells post treatment 0–48 hours, with BKM120 at 31.25 to 4000 nM, compared to vehicle cells.

**Fig 4:**
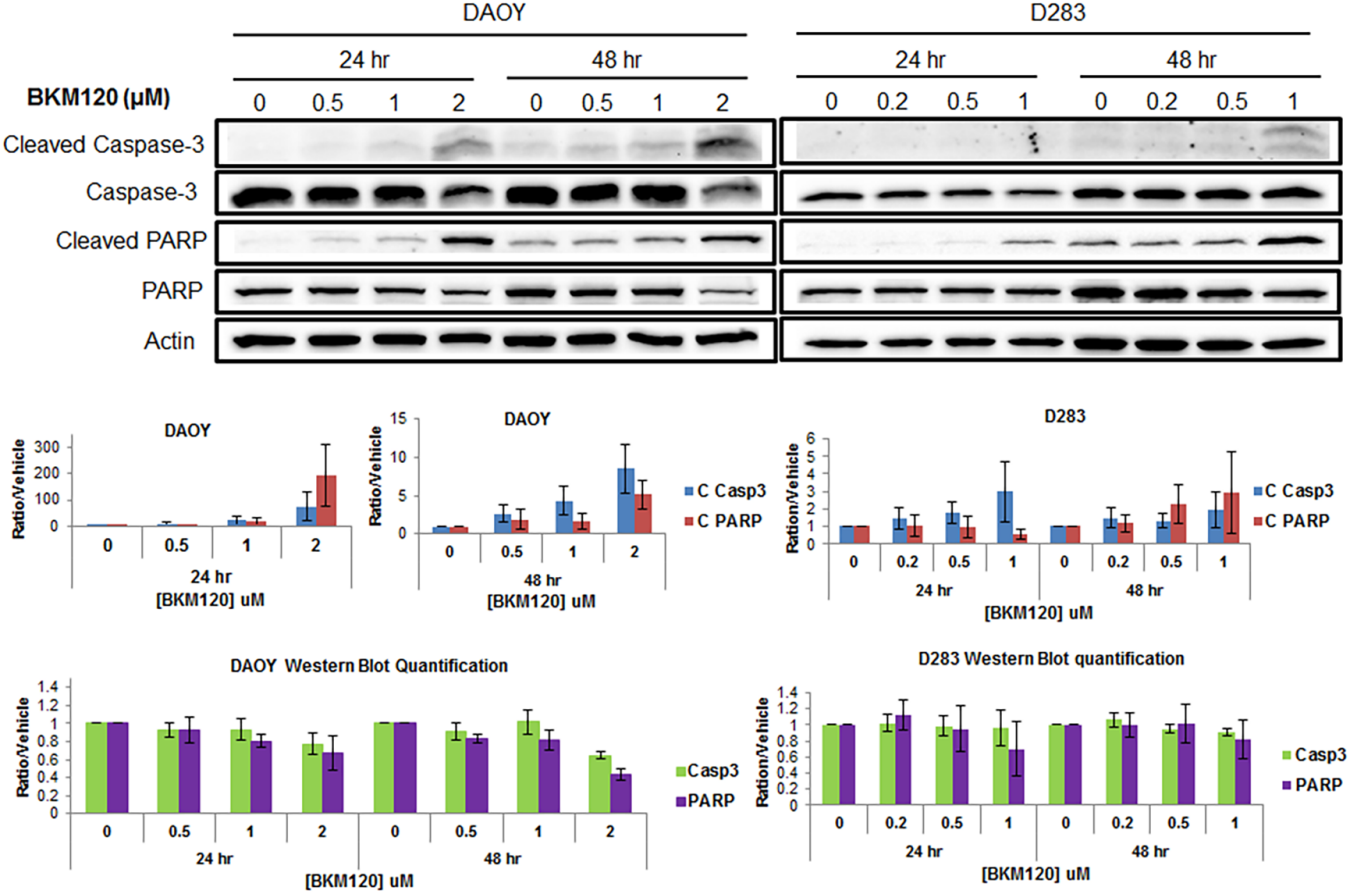
BKM120 induces apoptotic markers in MB cells. MB cells were treated with three concentrations of BKM120 for 24 and 48 hrs. Cell lysates were collected and western blot analysis was performed to measure Cleaved Caspase-3 and cleaved PARP. Level of total Caspase-3 and PARP were also examined. Actin was used as overall loading control. Optic density of bands were quantified using Image Lab Software (Bio-Rad). The relative band intensities of Cleaved Caspase-3, Cleaved PARP, total Caspase-3 and total PARP were normalized to actin and vehicle control (value = 1).

To determine if BKM120 induces apoptosis in MB cells, DAOY, D283 and D384 cells were treated with BKM120 ranging from 0 to 4 μM for a time lapse of 48 hours. IncuCyte ZOOM Live-Cell Imaging system was used for kinetic monitoring of cytotoxicity and apoptosis of the treated cells, in the presence of Caspase 3/7 Apoptosis Assay Reagent. Fluorescent images were collected every 4 hours. At 48 hour post treatment, representative images indicated that BKM120 induced apoptosis in MB cells is dose-dependent (Fig 3A). Quantification of apoptosis (ratio of green object count/well of caspase 3/7 versus vehicle) over a time lapse of 48 hours showed BKM120 induced caspase-mediated apoptosis in MB cells in a dose-dependent manner over time in the three cell lines tested, DAOY, D283 and D384 (Fig 3B). Western blot analysis also confirmed that BKM120 induces apoptosis in MB cells. DAOY and D283 cells were treated with three concentrations of BKM120. Cell lysates were collected at 24 and 48 hours. Cleaved Caspase 3 and cleaved PARP levels increased in the cells treated with BKM120 in a concentration dependent manner, confirming the apoptosis assay data obtained from IncuCyte ZOOM (Fig 4).

### BKM120 deactivates AKT/mTOR and MAPK pathways

**Fig 5:**
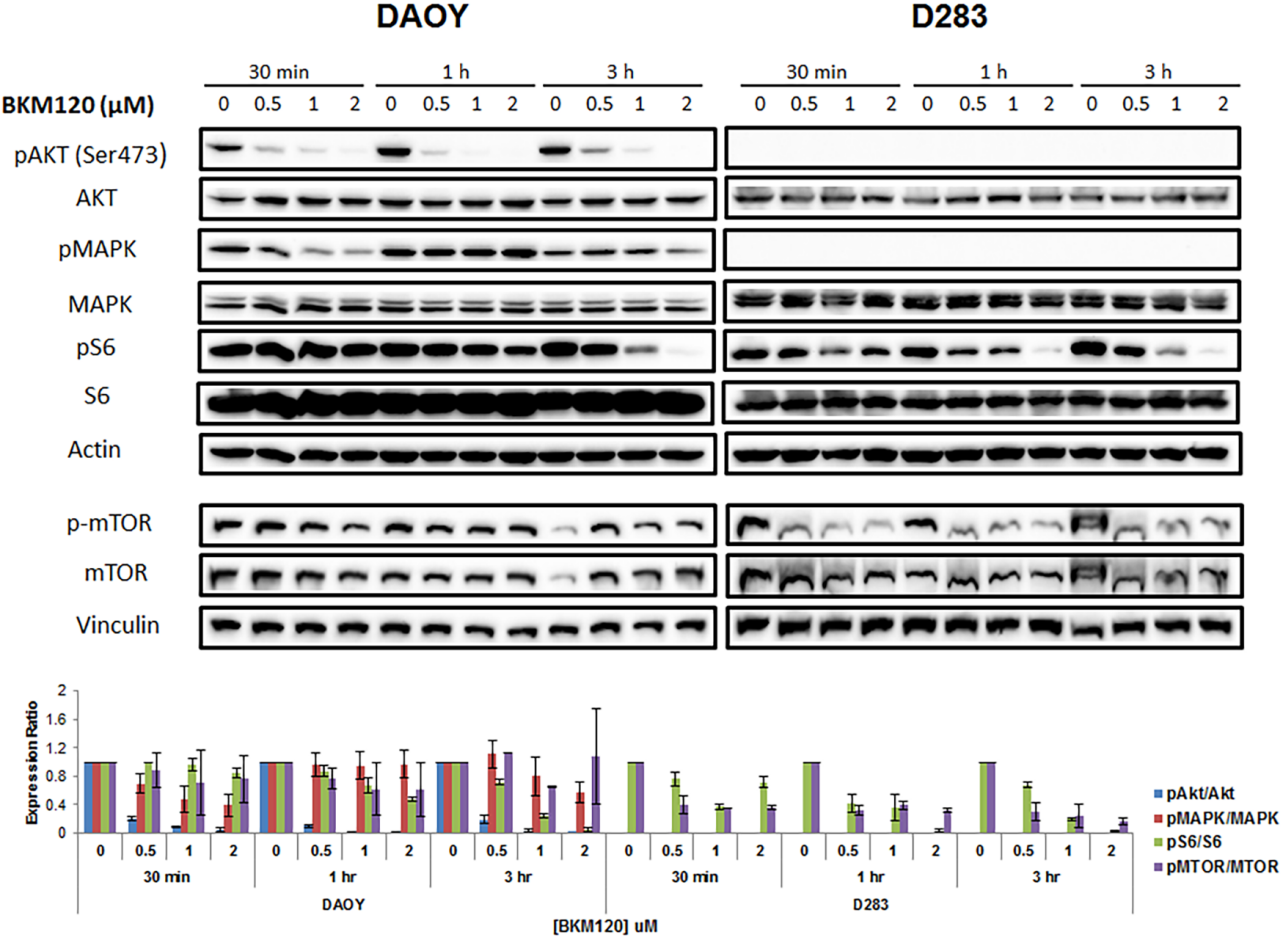
BKM120 inhibits phosphorylation of downstream molecules. MB cells were treated with 0.5, 1 and 2 μM BKM120 for 30 min, 1h and 3 h. Cell lysates were collected and western blot analysis was performed to measure pAKT, pS6, pMAPK, p-mTOR, total AKT, S6, MAPK and mTOR. Actin was used as overall loading control. The Western blots were repeated at least 3 times. Optic density of bands were quantified using Image Lab Software (Bio-Rad). The relative band intensities of pAKT, pMAPK, pS6 and mTOR were calculated against the total protein (AKT, S6, MAPK, and mTOR), and values were normalized to control (value = 1).

Next we examined how BKM120 affects downstream activities. DAOY and D283 cells were treated with three concentrations of BKM120 (0.5, 1 and 2 μM) for various time points (30 min, 1h, and 3h), and active AKT, MAPK (ERK1/2), mTOR, and S6 kinase (a direct substrate of AKT) levels were examined. Western blot analysis was performed using whole cell lysates. BKM120 treatment reduced S473P-AKT in DAOY cells in a dose-dependent manner (Fig 5). pAKT inhibition was observed within 30 min of BKM120 treatment and it remained inhibited at least for 3h. Similarly, S2448P-mTOR levels were reduced when both cell lines were treated with 0.5, 1 and 2 μM of BKM120, at all time points examined (Fig 5). Inhibition of pAKT and mTOR was followed by a decrease phosphorylation of its downstream target S6 Ribosomal Protein in a BKM120 dose-dependent manner. Interestingly, BKM120 treatment inhibited pMAPK levels in DAOY cells in a dose-dependent manner (Fig 5). Inhibition of MAPK phosphorylation was observed at 30 min then recuperate to basal levels at 1h followed by inhibition again at 3h suggesting a transient and cyclic effect of BKM120 on MAPK activity. The results were further confirmed by quantification of relative band intensities.

Endogenous pAKT and pMAPK levels were very low in D283 cells (*TP53* wild type) (Fig 5). Therefore the effect of BKM120 on these molecules were inconclusive for this cell line. Interestingly, BKM120-induced deactivation of S6 Robosomal Protein was observed in D283 cells although we could not detect pAKT in this cell line. This suggests that there must be residual levels of pAKT which was inhibited by BKM120.

### BKM120 decreases cellular glycolytic metabolic activity in MB cell lines

**Fig 6:**
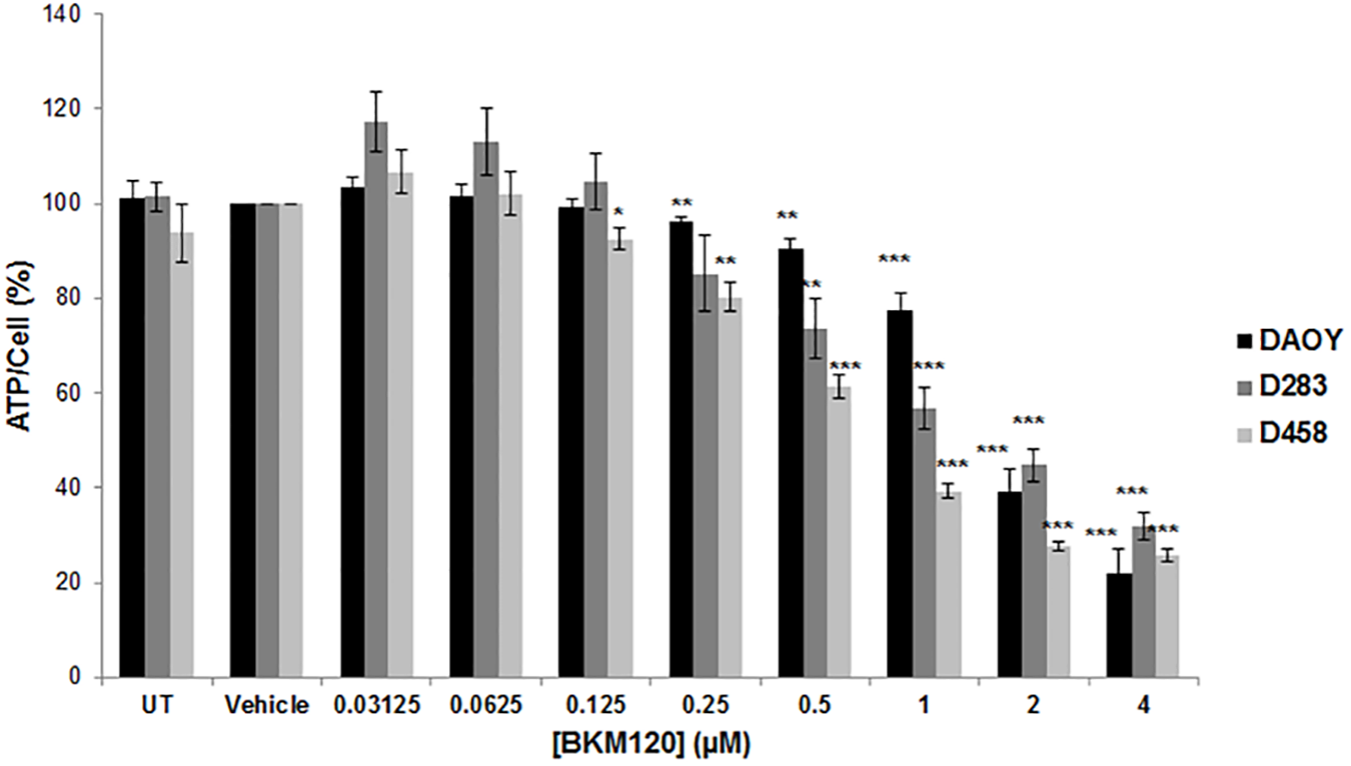
BKM120 decreases cellular glycolytic metabolic activity in MB cell lines. MB cells were treated with BKM120 ranging from 31.25 nM to 4 μM over a course of 48 hours. ATP/cell assays showed a decrease in ATP level per cell after 48 hours of BKM120 treatment, which indicates a decrease in cellular glycolytic metabolic activity in MB cell lines in a dose-dependent manner. Data are presented as percent of vehicle control. *p < 0.05; **p < 0.01; ***p < 0.001.

Given the role of the PI3K/AKT/mTOR pathway in the regulation of cell metabolism, we examined the cellular ATP levels in BKM120-treated cells. The CellTiter-Glo Luminescent Cell Viability Assay is a homogeneous method of determining the number of viable cells in culture based on quantitation of the ATP present, an indicator of metabolically active cells. Dividing CellTiter-Glo data by CyQuant data (measure of cell number) and normalized to vehicle control gave rise to percent ATP level per cell. BKM120 decreased ATP level/cell in the three cell lines tested DAOY, D283 and D458, in a dose-dependent manner (Fig 6), indicating that BKM120 inhibits cellular glycolytic metabolism in MB cells.

### BKM120 displays strong antitumor activity in tumor-bearing mice and prolongs survival

**Fig 7:**
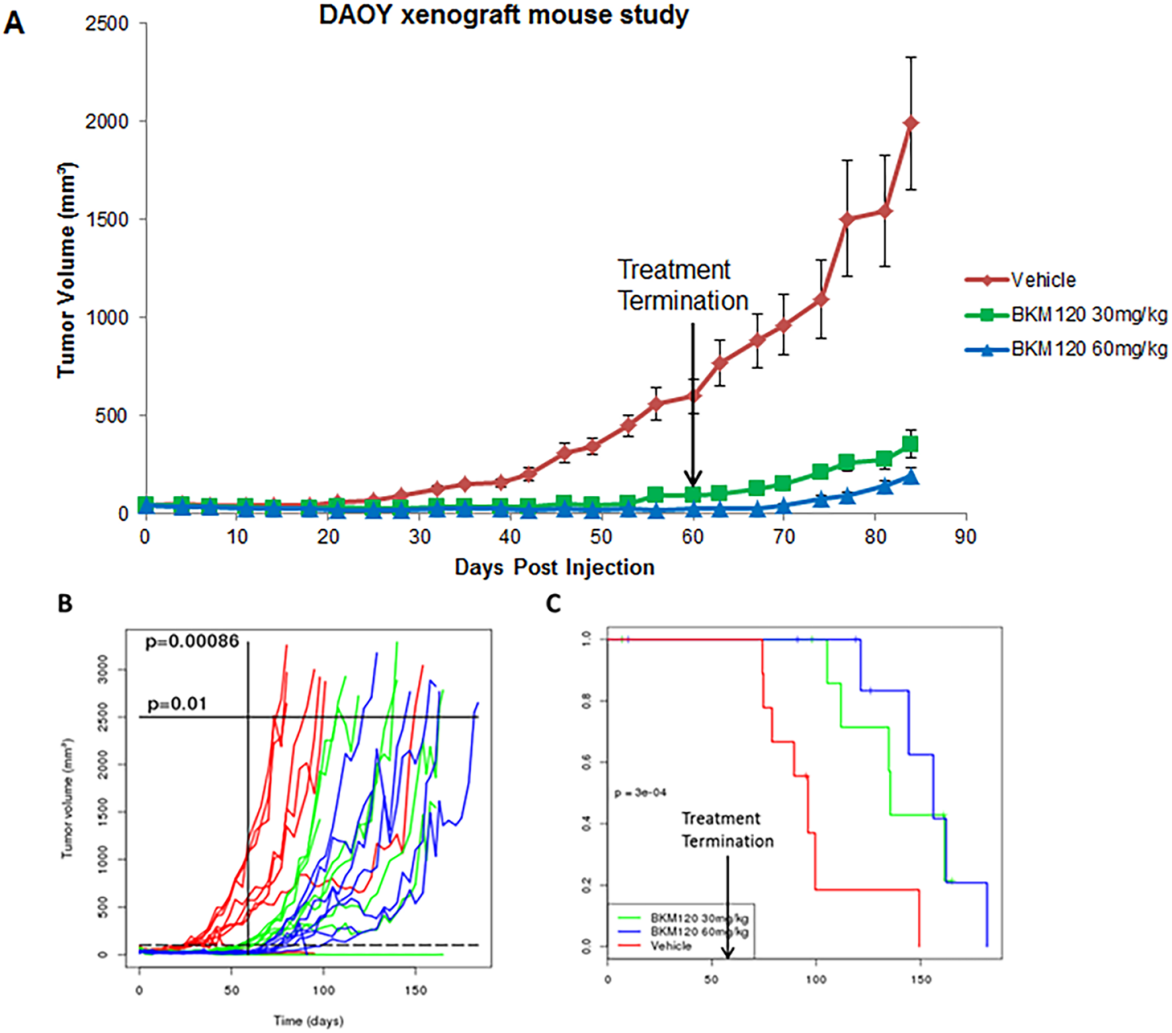
BKM120 displays strong antitumor activity in tumor-bearing mice and prolongs survival. (A) BKM120 inhibits tumor growth in DAOY MB xenograft mouse study. Group 1: Vehicle control; Group 2: BKM120 at 30 mg/kg; Group 3: BKM120 at 60 mg/kg. BKM120 treatment was administered by oral gavage daily for 60 days (n = 9 per group). (B) Growth curves of individual mouse in each group, with *p* value of 0.00086 on day 60; and with *p* value of 0.01 at tumor size of 2500 mm^3^. (C) Kaplan–Meier plot of the xenograft study. BKM120 significantly prolonged mouse survival in DAOY MB xenograft mouse study (*p* = 0.0003). Arrow indicates termination of treatment for all the groups. Experiments were repeated two times with 9 mice in each group.

To explore the anti-tumor efficacy of BKM120 in MB xenograft model, DAOY cells were injected subcutaneously in nude mice. When tumors became palpable, treatment began and BKM120 was administered by oral gavage daily at 30 mg/kg and 60 mg/kg for 60 days. During the treatment, tumor sizes in both BKM120 treatment groups remained minimal, while tumors in vehicle group grew steadily (Fig 7A). Even after the treatment was terminated at day 60, mice that were treated with BKM120 had a slow tumor growth rate whereas mice in vehicle treated group had a fast tumor growth rate (Fig 7A). Growth curve analysis of tumor volume revealed that BKM120 significantly suppressed tumor growth with *p* value of 0.00086 on day 60 (Fig 7B). Time-to threshold tumor volume of 2500 mm^3^ for BKM120 treated group was significantly higher than vehicle control group (p value of 0.01) (Fig 6B). Kaplan–Meier plot indicated that BKM120 significantly prolonged mouse survival in treated groups, with a *p* value of 0.0003 (Fig 7C). Experiment was repeated to confirm the results.

## Discussion

In this study, we examined the mechanisms of BKM120 in a panel of both established and patient-derived primary medulloblastoma cell lines and found that BKM120 exhibits a strong anti-tumor efficacy both *in vitro* and *in vivo*, through caspase-mediated apoptosis.

*TP53* is mutated in approximately 10% of MB and its impact on survival is controversial [2]. Opposing results have been reported in regards to sensitivity of cell lines to NVP-BKM120 treatment with respect to *TP53* status [28–30]. Koul et al reported that glioma cells containing wild-type *TP53* were more sensitive to BKM120 than cells with mutated or deleted *TP53* [28]. Whereas other studies have shown no obvious correlation between BKM120 sensitivity and *TP53* and/or PTEN status [29–31]. In our study, all MB cells were sensitive to BKM120, albeit moderately lower IC50 values of *TP53* WT cells (D384, D458, D283, D341, D487, D721 and D556) than those of *TP53* mutants (DAOY and D581). However, we need to examine more MB cell lines to clearly establish a correlation between BKM120 sensitivity and *TP53* status in MB. Koul et al reported that PARP and caspase-3 were only cleaved after BKM120 treatment in *TP53* WT cells but not in *TP53* mut/del cells [28]. In contrary, we found equal numbers of apoptotic cells, and a significant increase in cleaved caspase 3 as well as cleaved PARP in both *TP53* WT (D283) and *TP53* mutant (DAOY) MB cells in response to BKM120. Our results are in agreement with a recent report by Bashash et al. [30] in which they demonstrated that BKM120 induced apoptosis in both *TP53* WT (Nalm-6) and *TP53* mutant (NB4) acute leukemia cell lines. They also proposed that while BKM120 induced p53-dependent apoptotic pathway in Nalm-6, PI3K/Akt/NF-κB axis was inhibited in *TP53* mutant-NB4 cells. Although the mechanism is not fully understood, it is clear from our results that BKM120 induced both p53-dependant and p53-independent cell death in MB cells. It is interesting that our patient-derived primary cell lines (BIO-054-08, BIO-074-08 and BIO-094-08) did not have lower IC_50_ values as the *TP53* WT established lines did. The exception might be due to the fact that they do not proliferate at the same rate as the established lines do. They were low-passage MB patient cell lines and did not grow well and fast enough for other *in vitro* and mouse studies. More MB patient primary tumors would be needed for further investigation to generate conclusive results as Gillet et al summarized in their work of The Clinical Relevance of Cancer Cell Lines [32].

Although we tend to detect higher levels of endogenous AKT phosphorylation in DAOY cells harboring a *TP53* mutation, BKM120 treatment efficiently inhibited overall AKT/mTOR pathway in both MB cells. We also evaluated MAPK status since inhibition of the PI3K pathway frequently leads to activation of alternate pro-survival signaling pathways [33, 34], and aberrant RAS/MAPK pathway has been implicated in the development of medulloblastoma [35]. The alternate activation of PI3K/AKT/mTOR and RAS/MAPK often led to single agent resistance in preclinical models [34, 36]. Remarkably, we found deactivation MAPK by BKM120, albeit reversible manner. A similar finding was observed by Mohan et al. [37] where rapamycin treatment caused reversible MAPK downregulation in MB cells. Our results also suggest that BKM120 inhibited glycolytic pathway as seen by dose-dependent decrease in ATP levels. Increased glycolysis and ATP production is the predominant pathway by which AKT activates mTOR and tumorigenesis [38]. Recent studies have shown cancer stem cells rapidly produce ATP via glycolysis [39] and targeting this pathway has been an important consideration to prevent progression, relapse, and resistance to chemotherapy and radiation. Interestingly, Frasson et al and Singh et al recently reported that PI3K inhibitors preferentially target medulloblastoma cancer stem cells (CSCs) [26, 40]. Singh et al exclusively examined CD15+ sub-population of one patient-derived SHH MB [26]. Given the fact that DAOY cell line belongs to SHH subgroup [41], our mouse study results supported their conclusions.

In MB xenograft study, treatment of BKM120 was terminated at day 60, at which point there was still no sign of growth in the treatment groups. The *p* value between vehicle and treated groups at day 60 is 0.00086, indicating a significant difference in tumor volumes. We observed slow tumor growth rate in BKM120 treated group even after discontinuation of the drug. As a result the tumor volume was significantly smaller in BKM120 treated group when the control group reached threshold tumor volume of 2500 mm^3^. These results suggest that the beneficial effect of BKM120 on reducing tumor growth persists even after the treatment regime is over. This observation is further supported by our finding that event free survival rate is markedly increased in BKM120 treated mice as compared to controls. There were a few reports examining the effect of BKM120 on tumorigenesis in mice. Buonamici et al explored mechanisms of a Smo antagonist LDE225 in medulloblastoma in 2010 [24]. They used BKM120 for a combination treatment with LDE225 in Ptch+/− p53−/− derived medulloblastoma allograft studies. However, BKM120 had no effect on tumor growth as single agent at 30 mg/kg. In our study, BKM120 at 30 mg/kg significantly inhibited tumor growth and prolonged survival. The compound seemed to be well tolerated by the animals, during prolonged treatment of 60 days. The differential observation maybe due to different mice models.

BKM120 can cross blood-brain barrier, making it an attractive option for the treatment of MB. Both subcutaneous and orthotopic xenograft models have been used in MB studies [42–44]. Subcutaneous models are suitable for initial drug testing as this model allows for easy tumor visualization, making decisions of treatment initiation and drug application less difficult [45]. Orthotopic models of MB requires additional features such as modification of MB cells to stably express luciferase gene in order to visualize tumor growth, but this model may provide additional information. Patient derived xenograft and orthotopic models for MB are currently under development in our laboratory for future animal studies. Although BKM120 is currently being evaluated in clinical trials in patients with a number of solid tumors, it has not been evaluated clinically in medulloblastoma.

It has been reported that BKM120 combined with irradiation enhanced the apoptosis of hepatocellular carcinoma cells [46]. PI3K/AKT/mTOR pathway inhibition can also sensitize cancer cells to chemotherapy [47], providing a rationale for BKM120 and chemotherapy combinations. BKM120 in combination with IGF1R inhibitor AEW541 and mTOR inhibitor rapamycin have been evaluated in pediatric bone and soft tissue sarcomas [48]. Novartis-sponsored and Novartis-supported trials of BKM120 in combination with other therapeutics in a range of cancer types are summarized by Massacesi et al [49]. Study of BKM120 in combination with chemotherapy and targeted compounds in medulloblastoma is warranted and underway in our laboratory, with a goal of evaluating them in clinical trials.
